# Implementation fidelity of the Systems for Person-Centered Elder Care (SPEC): a process evaluation study

**DOI:** 10.1186/s13012-021-01113-3

**Published:** 2021-05-12

**Authors:** Hyoungshim Choi, Young-il Jung, Hongsoo Kim

**Affiliations:** 1 Department of Nursing, Hansei University, 30 Hansei-ro, Gyeong-gi, South Korea; 2 Department of Environmental Health, Korea National Open University, 86 Daehak-ro, Jongno-gu, Seoul, South Korea; 3 Graduate School of Public Health, Department of Public Health Sciences, Institute of Aging, Institute of Health and Environment, Seoul National University, 1 Gwanak-ro, Gwanak-gu, Seoul, South Korea

**Keywords:** Process evaluation, Geriatric care model, Long-term care, Evaluation, Technology

## Abstract

**Background:**

The Systems for Person-Centered Elder Care (SPEC), a complex intervention, was conducted to examine its effectiveness as a technology-enhanced, multidisciplinary, and integrated care model for frail older persons among ten nursing homes (NHs) in South Korea where formal long-term care has recently been introduced. The purpose of this study was to evaluate the implementation fidelity of the SPEC intervention and to identify moderating factors that influence the implementation fidelity.

**Methods:**

This study was a process evaluation based on an evidence-based framework for implementation fidelity using a mixed-methods design. Quantitative data from consultant logbooks, NH documentations, an information and communications technology (ICT) system, and a standardized questionnaire were collected from April 2015 to December 2016 and analyzed by calculating the descriptive statistics. Semi-structured focus group interviews were held with multidisciplinary teams from the participating NHs. Qualitative data from a semi-structured questionnaire and the focus group interviews were analyzed using content analysis.

**Results:**

The SPEC program demonstrated good implementation fidelity, and adherence to the SPEC program was strong in all aspects, such as content, coverage, frequency, and duration. Of the participating on-site coordinators, 60% reported that the SPEC model positively impacted needs assessment and the reporting system for resident care. The important facilitating factors were tailored facilitating strategies, assurance of the quality of delivery, and recruitment strategies.

**Conclusion:**

The effectiveness of the SPEC program was driven by good implementation fidelity. The key factors of good implementation fidelity were tailored delivery of evidence-based interventions over process evaluation work, facilitating strategies, and ICT support. Larger implementation studies with a more user-friendly ICT system are recommended.

**Trial registration:**

ISRCTN registry, ISRCTN11972147. Registered on 16 March 2015

Contributions to the literature

- This study has shown that pre-planned theory-based process evaluation and a specified study protocol are important for implementation fidelity in complex interventions.
- This study addresses tailored strategies to facilitate implementation, such as consulting tailored to the needs and context of each participating nursing home, that are essential to increasing adherence in an intervention.
- We found that the cloud-based online ICT system improves the quality of care by promoting communication between care teams and the research team and generating useful institutional data to monitor and support resident care.

## Background

The quality of care for older residents in nursing homes (NHs) is closely related to their quality of life because the care is provided where they live [1–3]. If older adults residing in NHs do not receive quality services, they may experience poor health and functionality, more emergency room visits, unnecessary acute hospital admission, and premature death; these outcomes add to the burdens of older adults, families, and society as a whole [4, 5]. While innovative interventions to improve nursing home quality are valuable and much needed, such interventions for vulnerable older residents are often complex and difficult to implement [6].

Moreover, evaluating the effectiveness of innovative public health interventions is also challenging. Problems targeted by such interventions are associated with multiple causes targeting multiple levels, so the public health interventions include multiple components that can interact with one another, affecting both the respective and overall outcomes [7, 8]. Therefore, when evaluating a complex intervention study, it is important to understand the complex nature of interventions and to combine the outcome evaluation and process evaluation [7]. Effectively combining them is accomplished by focusing on the background elements that impact how an intervention’s goals are achieved, as these elements may also be applied when implementing the same intervention in other settings [7, 9]. Careful evaluation of contextual factors related to the intervention process helps identify the important functions of an intervention, even though complex interventions may seem uncontrollable due to various related phenomena [10–12].

Carroll et al. [13] proposed a conceptual framework for implementation fidelity (CFIF), which focuses on various aspects of adherence and moderating factors influencing intervention adherence. Several key components of CFIF are unique and valuable for assessing implementation fidelity: *facilitation strategies* such as intervention manuals, guidelines, training, and feedback are used to optimize and standardize how an intervention is conducted [13]. The *quality of delivery* is an assessment of whether the intervention delivery process is appropriate for achieving the original intentions of the intervention [13]. Quality of delivery is a significant potential moderating factor for the relationship between an intervention and its implementation fidelity [13]. The *responsiveness of participants* relates both to the persons who receive the intervention and to the persons who deliver the intervention [13]. The *intervention complexity* is also an important component of implementation fidelity, and it contributes to both the real nature of the intervention and the description of the intervention [13]. The more complex the intervention, the harder it is to obtain a higher level of implementation fidelity; however, if an intervention is described in great detail, it may enhance implementation fidelity [13, 14]. Additionally, CFIF assumes that an intervention delivered by a person with enthusiasm will have higher implementation fidelity [13]. Hasson [10] presented a modified CFIF by adding *context* and *recruitment* to Carroll et al.’s model, described above [13, 15]. *Contex*t refers to encompassing social systems that include structures and cultures of organizations and historical and concurrent events [15]. When implementing a complex intervention, organizational culture and the role of upper management are very important [16]. Both the older residents and the staff in NHs can experience optimal dignity, well-being, and health through a safe and caring organizational culture [17]. *Participant recruitment* includes reasons for nonparticipation, the consistency of recruitment procedures, and judgments by participants regarding the outcomes and relevance of interventions [18]. Hasson’s modified CFIF suggested using a systematic evaluation of all moderating factors, as the interactions between moderating factors are known to affect the implementation of an intervention [15, 18]. Hasson’s modified CFIF has been empirically examined as a useful conceptual framework to systematically evaluate implementation fidelity and possible moderating factors affecting implementation fidelity [15, 19, 20]. It is valuable to measure and analyze implementation fidelity by providing guidance in organizing the data collection of adherence and associated moderators in complex interventions [15].

**Fig. 1:**
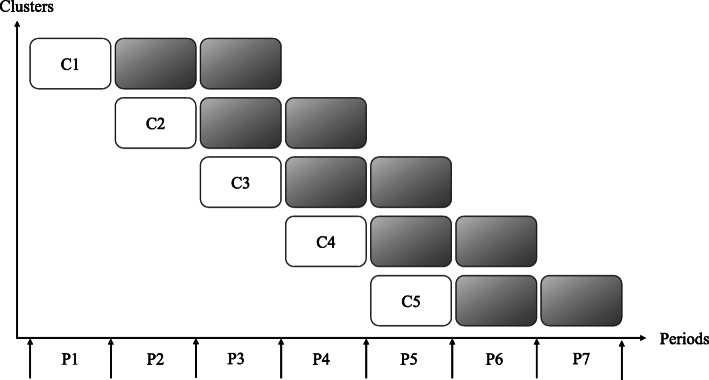
Design of the SPEC study. The study was an incomplete stepped-wedge cluster randomized control trial (Kim et al. [22]). Each of the five clusters contained two randomized nursing homes that implemented the SPEC program over the same period of time, from P2 to P7. The white cells represent the control units, and the gray cells represent the units where the program was implemented. The measures to generate incidence-based quality indicators were conducted at each arrow.

**Fig. 2:**
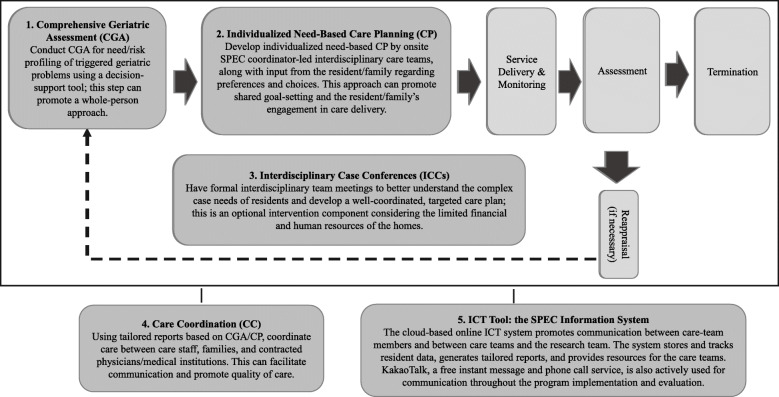
Diagram of the components and process of the SPEC program

The Systems for Person-Centered Elder Care (SPEC) is a technology-enhanced, multidisciplinary, integrated care model for older adults with frailty [9]. The intervention study hypothesized that the implementation of the SPEC model will improve the quality of care in NHs with limited healthcare provision, and the improvement will promote the health/functional outcomes and quality of life of old NH residents with multimorbidity [9]. This complex intervention was implemented in ten nursing homes that had been certified and reimbursed by public long-term care insurance (LTCI) in South Korea over 21 months between 2015 and 2016 (Fig. 1). As a complex intervention model based on Wagner’s Chronic Care Model, the SPEC [9] consists of five components: comprehensive geriatric assessment (CGA) based on the Korean version of the interRAI long-term care facility [21], individualized need-based care planning (CP), interdisciplinary case conferences (ICCs), care coordination (CC), and information and communications technology (ICT) tools (Fig. 2).

Each participating NH had an on-site SPEC coordinator team, typically composed of a nurse and a social worker, who performed CGA, developed a care plan from the assessment of each participating resident, ran optional ICCs, and communicated with the SPEC consultant. The SPEC consultant trained and empowered the NH staff, particularly the on-site SPEC coordinator team, to improve the care quality of the participating NHs. The SPEC consultant facilitated and evaluated the entire implementation process of the SPEC intervention for each participating NH; offered training sessions, care coordination, and on- and off-line consultation; and monitored the progress of the intervention through a cloud-based ICT system on a regular basis. The effectiveness of the intervention was evaluated through stepped-wedge cluster-randomized trials, and the intervention (the SPEC program) significantly improved quality of care as measured by a composite quality indicator (QI; mean difference −0.025 [CI −0.037 to −0.014, *p* < .0001]) [22].

**Fig. 3:**
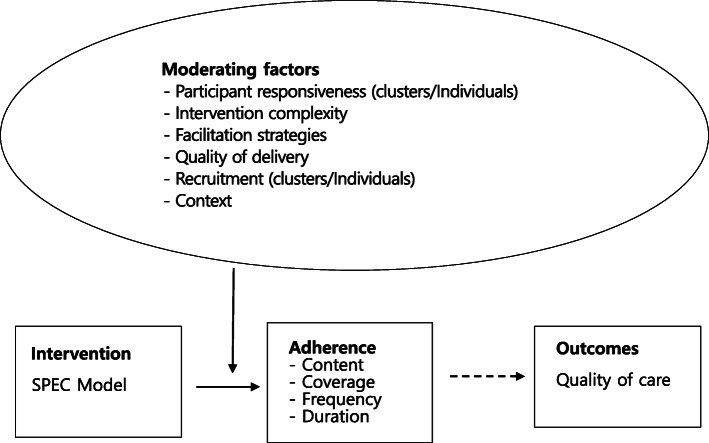
Conceptual framework of the study based on Hasson’s [15] and Grant et al.’s [23] framework for implementation fidelity

Kim et al.’s [22] study focused on evaluating only the effectiveness of the SPEC intervention; the process evaluation described in this article was conducted in parallel with the effectiveness study. We designed this study to evaluate implementation fidelity and to identify moderating factors that could influence the implementation fidelity of complex interventions. The conceptual framework of the current study (Fig. 3) was developed by combining Hasson’s [15] modified CFIF with Grant et al.’s [23] framework for designing process evaluations for cluster-randomized trials of complex interventions [15, 21]. The latter includes domains (e.g., recruitment of clusters, recruitment, and reach of individuals) that can help to investigate the external validity of an effectiveness study for the SPEC intervention.

This study hypothesized that fidelity is influenced by six moderating factors in the combined/modified CFIF: participant responsiveness to SPEC interventions, the complexity of interventions, facilitation strategies, the quality of delivery, context, and participant recruitment (Fig. 3). We measured the adherence of implementation fidelity by rating the degree of executed and delivered intervention components of the SPEC model based on four subdomains (contents, frequency, duration, and coverage) of the modified CFIF. We also analyzed the moderating factors by using a deductive approach based on the six moderating factors of the combined CFIF. This study aims to evaluate the level of implementation fidelity and explore the mechanism and moderating factors to optimize implementation fidelity for SPEC interventions for frail older adults in Korean nursing homes.

## Methods

### Study design

The present study used a mixed-methods design to investigate the implementation fidelity of the Systems for Person-Centered Elder Care (SPEC) study [9]. The SPEC intervention study utilized a prospective, incomplete, stepped-wedge cluster-randomized trial to examine the effectiveness of the SPEC model among ten NHs in South Korea. The SPEC intervention was implemented with older adult residents in each participating NH for 6 months after a 3-month control period. The intervention was rolled out to each NH over five different intervals in sequence [22] (Fig. 2).

### Study setting and participants

The intervention study was conducted at 10 NHs that agreed to participate, all located in Seoul and the major provinces in South Korea. In Korea, universal public LTCI for older adults with a certain level of dependency was newly introduced in 2008 in response to the changing family and social context and norms [24]. All the participating NHs had residents registered in and reimbursed by the public LTCI and met certain regulations for staffing, physical facilities, operations, and accreditation [25]. We recruited NHs with 30 or more residents and that met the staffing requirements of the LTCI: at least one registered nurse or nursing assistant per 25 residents, one personal care assistant per 5 people, and one social worker [26]. Older residents who received intervention were those aged 65 or older who had stayed in the participating NHs for at least 1 week, were neither in a terminal condition nor comatose, and were capable of study participation [22].

### Data collection

The overall process evaluation plans for the SPEC trial [9, 21] were based on the conceptual framework of this study addressed earlier (Fig. 3), guided by Hasson’s [15] modified CFIF and Grant et al.’s [23] framework model for designing process evaluation for cluster-randomized controlled trials [9, 15, 22, 23]. In each cluster, the process data were gathered right before, during, and after the 6-month intervention phase. Quantitative data measured were entries from the SPEC consultants’ logbooks, the SPEC ICT system, and standardized questionnaire items. Qualitative data were collected from the SPEC consultants’ logbooks, a semi-structured questionnaire for free-text responses, and focus group interviews. The questionnaires were 12-item self-reported paper questionnaires with two parts. One was a seven-item standardized questionnaire with a 5-point Likert scale, and the other was a five-item semi-structured questionnaire for free-text responses. The research team sent the questionnaire by e-mail to on-site SPEC coordinators and collected the completed questionnaires sealed in an envelope during the visit for the focus group interview. The standardized questionnaire and the results are reported in Table 3. Additionally, relevant data for process evaluation were obtained from the SPEC ICT system.

The focus group interviews with each NH’s interdisciplinary team were conducted by the first author, assisted by a research assistant for process evaluation after 6-month intervention periods for each NH for 1 h. Each interdisciplinary team consisted of four to eight professionals, including a NH head, practice nurse(s), social worker(s), physical therapist, occupational therapist, and nutritionist, as well as on-site SPEC coordinators. The focus group interviews were audio-taped and transcribed by two research assistants. The researchers carrying out the focus group interviews completed a post-interview reflection after each interview, which identified any issues regarding delivery. The data were used to evaluate adherence and potential moderators that affected the implementation fidelity of the SPEC model.

### Data analysis

Quantitative data from the standardized questionnaire and data from the SPEC ICT systems were analyzed with SAS 9.4 to measure descriptive statistics (frequency, means, and percentages). All qualitative data, including free-text responses from semi-structured questionnaires and focus group interviews, were analyzed using content analysis. The focus group interviews were audio-taped, transcribed, and coded. The coding framework, including domains of adherence and moderating factors guided by modified/combined CFIF (Table 1, Fig. 3) [13, 15, 22], was generated. The first author of this study carried out the coding of all qualitative data, and units of meaning within the text were allocated to each code. The corresponding author cross-checked the coding for inter-subjectivity. They then discussed until a consensus was reached. Themes regarding potential moderating factors that affected implementation fidelity were derived from the interviewees based on the theoretical framework from Hasson [15] and Grant et al. [23].

**Table 1:** Summary of the evaluation aspect data collection methods based on the conceptual framework guided by Hasson [15] and Grant et al. [23]

Theoretical elements (area to measure)	Research questions	Data source and data collection methods				
		LB	ICT	SQ	SSQ	FGI
**Evaluation of adherence**						
Content	- Was each of the intervention components implemented as planned?	x	x	x		
Frequency/duration (dosage, dose delivery)	- Were the intervention components implemented as often and for as long as planned?	x	x	x		
Coverage (reach)	- What proportion of the target group participated in the interventions?	x	x	x		
**Potential moderating factors**						
Participant responsiveness						
Clusters	- How was the intervention adopted by cluster? - Why did clusters agree to participate or not?	x				
Individuals	- How were the participants engaged with the interventions? - How satisfied were the participants with the interventions? - What were the barriers and facilitators to implement the interventions?	x		x	x	x
Intervention complexity	- How complex was the intervention? - How specific was the intervention description?	x		x	x	x
Strategies to facilitate implementation	- What strategies were used to support implementation? - How were these strategies perceived by staff involved in the interventions?	x			x	x
Quality of delivery	- How was the quality of delivering the intervention components?	x		x	x	x
Recruitment						
Recruitment of clusters	- How were clusters sampled and recruited?	x				
Recruitment and reach-in of individuals	- What recruitment procedures were used to attract individuals to intervention? - What constituted barriers to maintaining involvement of individuals?	x	x	x	x	x
Context	- What factors at political, economic, organizational, and work focus group levels affected the implementation?	x		x	x	x

*LB*, SPEC consultant’s logbooks; *ICT*, SPEC ICT system; *SQ*, standardized questionnaire items from on-site SPEC coordinators; *SSQ*, semi-structured questionnaire from on-site SPEC coordinators; *FGI*, focus group interviews with interdisciplinary team of each participating NH

## Results

### Adherence

#### Content

All intervention components were delivered to ten NHs as planned [9]. The SPEC consultant provided education and consultations regarding CGAs, individualized need-based CP, and optional ICCs, CC, and ICT tools for each on-site SPEC coordinator. With the support of the SPEC consultant, the actual SPEC intervention components were executed and delivered to participating residents by the care team led by an on-site SPEC coordinator team for each participating NH (Table 2). Using the ICT tool, the prototype SPEC ICT system, the research team provided tailored reports for NHs, families, and physicians who contracted with each participating NH. The research team operated the help desk to provide immediate support regarding the SPEC program.

**Table 2:** Summary of adherence of Systems for Person-Centered Elder Care (SPEC) model

Components	Number of planned interventions	Number of delivered interventions (%)	Providers
1. **Comprehensive geriatric assessment (CGA)**			
Delivered CGA education via in-service training	10	10 (100)	By the SPEC consultant with the research team
Performed individual CGA profiles	482	482 (100)	By the on-site SPEC coordinators at each participating NH
2. **Care plan (CP)**			
Delivered CP education via in-service training	10	10 (100)	By the SPEC consultant
Performed individual CP profiles	482	419 (86.9)	By the on-site SPEC coordinators at each participating NH
3. **Interdisciplinary case conferences (ICCs)**			
Performed ICCs with support	10	10 (100)	By the care team led by the on-site SPEC coordinators at each participating NH and facilitated by the SPEC consultant
Performed ICCs without support	50	50 (100)	By the care team led by the on-site SPEC coordinators at each participating NH
4. **Care coordination (CC)**			
Delivered tailored reports to NHs	10	10 (100)	By the SPEC consultant and research team
Delivered tailored reports to residents/families	482	419 (86.9)	By the on-site SPEC coordinators facilitated by the SPEC consultant
Delivered tailored reports to physicians	10	10 (100)	By the on-site SPEC coordinators facilitated by the SPEC consultant
5. **Information and communications technology (ICT) tools: the SPEC information system**			
Delivered ICT tools	10	10 (100)	By the on-site SPEC coordinators facilitated by the SPEC consultant

#### Frequency/duration (dosage/dose delivery)

All intervention components were provided for participating NHs with the originally planned frequency and duration. CGA, CP, and CC were delivered by the on-site SPEC coordinator teams (RN-SW pair) at each participating NH at least one time for each participating resident; CP was implemented for participating residents for about 6 months. ICC for complex cases was implemented at least once a month by the care team led by the SPEC coordinator team at each participating NH with or without the SPEC consultant. Additionally, the on-site coordinator team communicated with the SPEC consultant and the help desk staff whenever necessary through KaKao Talk, a free instant message and phone call service (an ICT tool, Table 2). The results of a survey conducted with on-site SPEC coordinators revealed that they had easy access to the internet at work and accessed the SPEC ICT system at least 1 or 2 days per week (Table 3).

**Table 3:** Usage and user opinion for SPEC model and ICT system from on-site SPEC coordinators (*n*=20)

Process evaluation questionnaire	Answer	*n* (%)
**Usage of SPEC ICT system**		
Is it easy to access the internet at work?	Yes	20 (100)
	No	0 (0)
On average, how many days did you access and use the SPEC ICT system per week?	1~2 days/week	9 (45)
	3~4 days/week	9 (45)
	≥5 days/week	2 (10)
Would you recommend using the SPEC ICT system for care providers of other NHs?	Yes	9 (45)
	Maybe	7 (35)
	No	4 (20)
Given the choice, will you increase or decrease your usage of the SPEC ICT system?	Increase	3 (15)
	Maintain	6 (30)
	Decrease	11 (55)
**User opinion about SPEC model**		
Did the SPEC model have a positive or negative impact on the need assessment and reporting system for resident care?	Positive	12 (60)
	Neutral	7 (35)
	Negative	1 (5)
How helpful was the SPEC model in terms of your care planning and evaluation for residents?	Helpful	13 (65)
	Neutral	6 (30)
	Not helpful	1 (5)
Was the adoption of the SPEC model helpful in reducing the amount of time to set up your care planning for residents?	Helpful	10 (50)
	Neutral	6 (30)
	Not helpful	4 (20)

#### Coverage (reach)

Participants also included 482 residents who received the SPEC intervention [22]. CP and CC were provided to 86.9% of the residents, while other intervention components were delivered to all participating NHs and residents as planned (Table 2). The SPEC consultant delivered the CGA and CP training to the on-site SPEC coordinator teams, and the NH staff members (including the coordinator team) performed the CGA and CP for participating residents. In every participating NH, a multidisciplinary care team performed at least six ICCs—one ICC per month for the 6-month intervention periods. The SPEC consultant and the on-site SPEC coordinator teams cooperated to conduct the first and second ICCs. A SPEC consultant led the first ICC to demonstrate the process to an on-site SPEC coordinator team in each home, and they conducted the second ICC with the help of the SPEC consultant. The remaining four ICCs were left to the on-site SPEC coordinator team at each home.

### Moderating factors

#### Participant responsiveness

There were positive responses from the participants regarding the SPEC model. About 75% of on-site SPEC coordinators answered that they would recommend the SPEC model to other NHs. However, only 15% were willing to increase the usage of the SPEC ICT system in their NH (Table 3). Positive responses from participants regarding the general SPEC model included boosting CGAs, facilitating communication, and providing tailored care plans. The participating on-site SPEC coordinators expressed that CGA was very helpful for assessing the needs of participating residents. They also addressed how the SPEC model (intervention) facilitated communication among multidisciplinary teams. Individualized needs-based CP was reinforced by patient-centered care. Most negative responses from participants focused on SPEC ICT system use regarding user-friendliness and additionally included duplication in documentation with the existing system and lack of staffing levels and time. The on-site SPEC coordinators complained that they had to use an existing system along with the SPEC system, leading them to consider the use of the SPEC ICT system to be duplicated work. Moreover, they felt overwhelmed because they had to carry out their duties within their regular work hours with extra time and staffing necessitated to accomplish all of the tasks related to the SPEC intervention (Table 4).

**Table 4:** Themes of moderating factors and selected interview quotations on experiences and perceptions of respondents in implementing SPEC model

Domain	Themes	Representative quotation from interviews
Participant responsiveness	Boosting CGA	“The CGA tool was very useful to see the images of the old adults at a glance and grasp the needs comprehensively.”
	Facilitating communication	“Communication between care teams was enhanced with the SPEC ICT system.” “At the case conference, care teams gathered and communicated about the patient, making it easier to understand the patient and get help from other teams to implement interventions for patients.”
	Providing tailored care plan	“The care plans are based on the list of problems derived from the needs assessment and therefore, enable us to promote individual approach and to provide tailored care plan for each patient.”
	Duplication of using SPEC system	“I consider it a duplication using both the SPEC ICT system and the system currently in use.”
	Lack of staffing level and time	“I know the SPEC model is good, but we don’t have human resources and time to use it. There is no time to feed the result of needs assessments and care plans into the SPEC model during working hours. If I want to use the SPEC model, I may have to work overtime.”
Intervention complexity	Comprehensiveness of intervention description	“The detailed manuals and materials for interventions were helpful, so I can handle the tasks even though the interventions were complex.
	Complexity of interventions	“The SPEC model has so many steps. If we make an individual care plan, we should complete CGA, prioritize the problem lists and choose interventions for each problem from intervention checklists and get consent for the care plan from patients or their family.”, “Needs assessment items are too long and hard to understand.”
	Difficulty of using ICT system	“The method of inputting into the SPEC ICT system is too complicated because most of the staff using the ICT system are not familiar with the computer.”
Facilitating strategies	Provision of immediate feedback	“The SPEC consultant and help desk provide immediate answers for any question asked through phone call and KakaoTalk, and it is very helpful.”
	Tailored consulting and extra education	“I had no clue when I had to organize a case conference for the first time, but it was very helpful of the SPEC consultant to participate in the case meeting, giving us feedback and providing us with advice on planning the intervention.”
Quality of delivery	Well-prepared training and manuals	“The training was well prepared overall, and after the theoretical training, it was good to have a practical training with the care team to evaluate the needs of real patients in the NH and set up care plans for the residents.”
	Provision of individual/institutional report	“The most valuable advantage is that the individual problem list is automatically derived from the needs assessment of each patient by algorithm, which is not included in any existing evaluation system.” “It was good to me that I can check the execution rate of intervention immediately through the SPEC ICT system.” “An institutional report on the list of problems and the number of drug use for a NH are very helpful.”
	Reflection of preferences of participants	“When I set up a patient’ care plan, I need to get consent about the care plan and therefore, ask the patient what he/she wants.” “In particular, if the patient is selected as a candidate for a case conference, we try to identify what interventions are particularly preferred by the patient and reflect them in the patient’s care plan.”
	Needs for practical system for Korean NHs	“It seems that some items in the CGA tool for the old adults do not fit with Korean NHs.” “When I am trying to make a care plan for an old adult, I don’t have enough contents to choose from in the care plan checklists of the SPEC ICT system. It would be better to have a more realistic and practical list of care plans for Korean NHs.”
Recruitment	Recruiting a proactive NH head	“We expect the SPEC model to be used systematically to evaluate needs and establish care plans.” “Even if it’s difficult, I think we should use the SPEC model to improve the quality of care of NHs.”
	Presentation for key care team for participation	“I cannot decide whether or not to use the SPEC model solely by myself even though I am a NH head, so I would ask the research team to visit my NH and explain it to the care team.” “My staff worried about new tasks adding burden on the participant, uncertainty about the effectiveness of interventions, and the potential to receive complaints from patients and their families due to participation in the study.”
	Difficulty of maintaining participation due to workload	“I don’t have enough time to use the SPEC ICT system. Some employees say that they would quit their job if I keep asking them to use the SPEC ICT system.”
Context	Supportive or individualistic organizational culture	“Multidisciplinary care team work together when we evaluate patient’s needs and set up care plans, making it easy to use the SPEC ICT system. For example, nurses, social workers, and physical therapists each take care of certain items given in the assessment tool.” “The nursing team does not want to get involved in using SPEC ICT system at all and does not cooperate, either.”
	Supportive leadership	“The staff seems to be working hard in using the SPEC ICT system because at the weekly meetings held each Monday, the NH head emphasizes the need for the SPEC model and the quality improvement, and also checks the execution rate of the intervention checklist.”
	Current events (Evaluation of National Health Insurance Services, resignation of key person etc.)	“I hope to delay the use of SPEC ICT system to after the institutional evaluation of the National Health Insurance Services.” “It is difficult to make an individualized care plan for each patient when the staff, who is in charge of setting up care plans with the SPEC model, quits the NH.”

#### Intervention complexity

The SPEC model is an integrated system with five interactive components. Each intervention component has unique steps and tasks. For example, an individual care plan needs a CGA for a single resident and a consent from the resident or family for the care plan. Sixty-five percent of on-site SPEC coordinators reported that the interventions were helpful for their care planning. However, only half of the on-site coordinators answered that their time needed for care planning was reduced and that the SPEC model made their care planning for NH residents easier (Table 3). Some on-site SPEC coordinators were satisfied with clear descriptions of interventions and their detailed manuals and materials. However, other on-site coordinators complained about the complexity of the ICT-based interventions. Some also found the detailed assessment items of the CGA tool difficult to fully comprehend. Additionally, the structured CP procedure through the ICT system was also considered to be somewhat challenging, as they were used to the paper-based, open-text style CP. Another major complaint reported was that the proto-type SPEC ICT system was not user-friendly. Completion of the computerized CGA and CP forms was challenging for most of the on-site coordinators, who were not familiar with electronic health recording systems (Table 4).

#### Strategies to facilitate implementation

General and tailored strategies were applied to facilitate implementation. General strategies included information sessions for top-level administers of participating NHs, a kick-off meeting for interventions for each participating NH, incentives for each NH, regular monitoring of the extent of intervention implementation through the ICT system, and provision of immediate feedback from the SPEC consultant regarding any questions about the SPEC model. Tailored strategies included motivational counseling with on-site SPEC coordinators, provision of extra education sessions, and consulting tailored to the needs and context of each NH. The SPEC consultant was responsible for facilitating and monitoring the implementation process. As a trained research nurse, the SPEC consultant neither visited the participating nursing homes daily nor conducted the intervention. The SPEC consultant was a type of circulating resource nurse coaching for implementation by the care staff team at the participating homes under the lead of the on-site SPEC coordinators; the main roles of the consultant were educating the on-site SPEC coordinators to conduct the CGA and make care plans, demonstrating how to use the computerized SPEC ICT system, coaching the care team to conduct case conferences, and monitoring the progress of the implementation process [9, 22]. Most on-site SPEC coordinators reported that the immediate responses of the SPEC consultant and the help desk when coordinators had SPEC model-related questions were very helpful, promoting the use of the SPEC ICT system and their maintained participation in this study. Furthermore, an on-site SPEC coordinator said that the extra education and counseling regarding case conferences enabled them to organize their own case conferences without needing the SPEC consultant (Table 4).

#### Quality of delivery

Approximately 65% of on-site SPEC coordinators answered that the SPEC model was helpful to change their needs assessment and reporting system; only 5% replied that the model did not help them with their care planning and evaluation for NH residents (Table 3). Most on-site SPEC coordinators rated the SPEC interventions as excellent. For example, the provision of individual/institutional reports—such as CGA-based need/risk profiling and individualized needs-based CP using standardized care protocols and checklists—promoted communication among contracted physicians, participating residents, and their families and enhanced implementation fidelity. They also reported that they were satisfied with the well-prepared training sessions and manuals, particularly the practical training with real cases from the NH directly following CGA or CP education sessions. They admitted that the process of agreement with individuals/families regarding goal setting and care planning facilitated reflection about the preferences or choices of participants. However, they also suggested that more user-friendly, practical ICT systems and protocols could be developed for use in Korean NHs (Table 4).

#### Recruitment

All of the residents who agreed and were eligible for this study in each NH received the SPEC intervention, and they were representative (Table 2). The research team recruited proactive NH heads who were interested in improving the quality of care. We gave presentations not only to NH heads but also to key care team members who could affect the decision of the NH heads regarding study participation. The barriers to participating and maintaining involvement were as follows: new tasks adding burden on the participants, uncertainty about the effectiveness of interventions, and the potential to receive complaints from patients and their families due to study participation (Table 4).

#### Context

Organizational culture was among the most important factors affecting implementation fidelity. The on-site SPEC coordinators who belong to NHs with a supportive and open organizational culture expressed that cooperation with other multidisciplinary teams encouraged them to overcome the difficulty of using the new system. On the other hand, an on-site SPEC coordinator who worked as a social worker for an NH with an individualistic and closed organizational culture reported that he had difficulties in implementing the SPEC model because some nurses of the nursing team were reluctant to use the SPEC ICT system. Supportive and proactive attitudes of the NH heads were also essential for successful intervention implementation. The shortage of long-term care workers in Korea has continued until recently; the shortage of nursing personnel is serious, and a high turnover rate is seen [25]. This is a factor that hinders the formation of a supportive, collaborative, and open organizational culture [25]. Additionally, unexpected events that required additional staffing and time negatively impacted aspects of the intervention implementation, such as initiating the accreditation process mandated by the public LTCI insurer. Public institutional evaluation for accreditation is mandatory every 2 to 3 years, and evaluation results are disclosed to the public, thus affecting users’ choices [26]. Implementation was also impacted in some cases by the resignation of a SPEC coordinator, a key person in intervention implementation (Table 4).

## Discussion

This process evaluation study aimed to investigate how the SPEC intervention, an ICT-enhanced, multidisciplinary, integrated care management model [9], was effective (positive outcomes) through examining the intervention’s implementation process. We found that the five components of the SPEC model were designed well for the purpose (quality improvement) according to the interviews with key participants (Table 4). We also examined how well the components were implemented as planned (Table 2). The strong adherence of participating homes—the implementation fidelity—was attributed to the moderating factors hypothesized in our conceptual model for this study. The importance of approaching implementation issues from a theoretical perspective and assessing fidelity within the process evaluation of a health program has been emphasized [19]. We were able to systematically evaluate adherence and moderating factors of implementation fidelity alongside the modified/combined CFIF. It was valuable to assess and analyze the roles of the six domains of the modified/combined CFIF. This is in line with previous studies emphasizing that the six moderating factors in the framework influenced fidelity in a complex and interrelated manner [18–20]. Tailored facilitating strategies, assurance of the quality of delivery, and recruitment strategies were facilitators. While intervention complexity was a barrier to adherence, the context and participant response both positively and negatively influenced implementation fidelity.

The main results of the SPEC study and the interviews with interdisciplinary team members (Table 4) supported the effectiveness of the five key components of the SPEC model. The health statuses of participating older residents were relatively severe and complicated; thus, those participants needed comprehensive and proactive interventions [27]. Therefore, the SPEC intervention required extra time and effort.

There were several facilitating factors for the successful implementation of the SPEC model. First is *tailored delivery of evidence-based comprehensive interventions over process evaluation work guided by the theoretical framework*. Pre-specifying and publishing study protocols improves the compliance of randomized trials of complex interventions by comparing intended interventions with implemented interventions [23, 28]. Previous studies suggested that consolidated framework and multi-faceted facilitating strategies are important for successfully implementing complex interventions [29, 30]. The SPEC research team developed theory-based process evaluations and followed the process evaluation protocols as much as possible (Table 2). According to a previous process evaluation study for dementia-specific case conferences in NHs, an optimizing process structure is very important for increasing the effectiveness of randomized controlled trials [31].

The second facilitating factor is *the role of the SPEC consultant and facilitating strategies*. The SPEC research team attempted to recruit proactive NH heads. We held presentations for key care teams because the expectations of the health care team for changes in the care process or improvement in the quality of care promote the performance of interventions [29, 30]. The SPEC coordinator provided well-prepared training and manuals to NH coordinators. In addition, the SPEC consultant monitored implementation fidelity through the SPEC ICT system regularly and gave immediate feedback to increase implementation fidelity. This finding correlates with previous studies suggesting that some actions of a facilitator, such as catalytic action in multidisciplinary teams and encouraging advice, influenced the effectiveness of the intervention positively [10]. Most on-site SPEC coordinators agreed that the tailored consulting was very helpful. A prior study’s finding that the support of a facilitator was the most attractive aspect of the nurse-led cognitive program regarding falls in older adults with frailty was consistent with the results of the current study [32].

The third facilitating factor is *the cloud-based online ICT system*, which supports resident care and generates institutional data. The ICT system was a prototype, computerized version of a CGA-based care management tool. Given the low staffing, the ICT system made it easier for the on-site coordinating team to implement the SPEC interventions (Table 3). The ICT system promoted communication between the care teams and the research team because they had access anytime and anywhere to on-site SPEC coordinators, who commented that the ICT system facilitated communication among multidisciplinary teams. The system improved the ease of storing and tracking resident data, generated useful tailored reports on the NHs, and provided resources for care providers/managers. Our findings were consistent with a previous study’s results indicating that e-consultation promotes data-driven improvements [33]. In this study, our research team was able to monitor the implementation fidelity in real time through the ICT system and manage the timeline for planned interventions for each participating NH. These findings resonate with a systematic review paper that e-consultation facilitates timely specialty advice [34].

In addition, several lessons to expand the SPEC model were learned. Firstly, sufficient institutional support is necessary to implement a new technology-enhanced integrated care model such as SPEC. Our results also highlight that high workloads and time pressure were the most widely represented barriers in applying new interventions in NHs [31]. According to the on-site SPEC coordinators, the SPEC model was useful for planning care and for improving the quality of care; however, participating NHs did not want to increase their usage of the SPEC model due to their heavy workloads and insufficient compensation (Tables 3 and 4). Therefore, sufficient workforce staffing and compensation through institutional and public LTCI policies for supporting institutions should be implemented to accommodate the adoption of a new system. Secondly, tailored and continuing education and training for NH staff are necessary for applying an ICT system in NHs. Via the user opinion questionnaire and interviews of key respondents, the participating staff members pointed out that they had difficulty using the SPEC ICT system and that the SPEC ICT system did not reduce the time required for effective care planning (Tables 3 and 4). According to a previous study with NH residents [35], heavy pressure to complete the interRAI-LTCF evaluation and insufficient computer equipment to perform it were hindrance factors [21]. Third, the addition of more specific and practical contents for participating NHs residents’ care is essential to promoting the performance of the interventions. In this study, some of the on-site SPEC coordinators asked for more practical and realistic care planning lists that apply to their residents (Table 4).

The SPEC study is considered to have been effective in achieving some expected results by conducting planned interventions, but it also has several limitations. First, the intervention and follow-up periods were short. The intensive intervention with the support of the research team for 3 months and the follow-up interval after the staff did not have the support of the research team for another 3 months was the minimum requirements for performance evaluation. If the intervention were to take place for a longer period, more NH residents would be able to participate in the optional ICC, and various effects might be observed. Second, although qualitative and quantitative assessments were used to understand the moderating factors for implementation fidelity, there may be missing subjects or areas. Moreover, we did not conduct a process evaluation of the responsiveness of residents and family members. Residents were likely to be unable to participate as they often had poor cognitive function, insufficient understanding of interventions, and difficulty with communication. The family members did not visit often and were not interested in being contacted for investigation. For this reason, this study mainly relied on the opinions of the service providers and did not investigate the resident experience and family/caregiver satisfaction. Third, the results of this study may not be objective because process evaluation was done by the SPEC research team. Fourth, there are limited studies of process evaluation on ICT-based quality improvement interventions that have been applied to clinical trials, making it difficult to compare this study with previous studies.

## Conclusions

The process evaluation study of moderating factors that affect the implementation fidelity of SPEC indicates that the effectiveness of the SPEC model may depend on whether or not the evidence-based interventions are strictly implemented. This study helps address the importance of process evaluation in promoting the implementation fidelity of interventions. It also provides evidence for developing theory-based process evaluations for adopting and diffusing a technology-enhanced care model for NH residents.

Therefore, this study offers several suggestions for future research. The development and application of research tools are required to reflect the responsiveness of participating residents with limited functional status and their family members, which will be helpful for comprehensive process evaluation. We also suggest a repeated study of SPEC on a large scale, under different settings, and in other countries. In addition, developing a more user-friendly ICT system can facilitate large-scale implementations of the SPEC program.

## Data Availability

The datasets generated by and/or analyzed during the current study are not publicly available due to the policy of the SNU IRB, which does not allow the opening and sharing of research data with any third party, but they are available from the corresponding author upon reasonable request.

## Abbreviations

CFIF: Conceptual Framework for Implementation Fidelity
LTCI: Long-term care insurance
CC: Care coordination
CGA: Comprehensive geriatric assessment
CP: Individualized need-based care planning
ICCs: Interdisciplinary case conferences
ICT: Information and communications technology
NH: Nursing home
SPEC: Systems for Person-Centered Elder Care
QI: Quality indicator
CI: Confidence interval
